# Supplementary material for: Neuroplastin genetically interacts with Cadherin 23 and the encoded isoform Np55 is sufficient for cochlear hair cell function and hearing
Source: PLoS Genet. 2022 Jan 31;18(1):e1009937. doi: 10.1371/journal.pgen.1009937 (PMC8830789; doi:10.1371/journal.pgen.1009937)
Supplement: S1 Fig — (A) Control data from Myo15-cre conditional cross compared alongside wild type Nptn+/+;Myo15-cre- mice (n = 5). Presence of the either the Myo15-cre allele (Nptnfl/fl;Myo15-cre+, n = 5), or the floxed Nptn allele (Nptn+/fl;Myo15-cre-, n = 7 or Nptnfl/fl;Myo15-cre-, n = 6) did not affect ABR thresholds. (B) Control data from the Prestin-CreERT2 conditional cross compared alongside wild type Nptn+/+;Prestin-CreERT2- with (n = 4) or without tamoxifen (n = 3). Mice dosed with tamoxifen had comparable ABR thresholds to those dosed with vehicle only. Presence of the Prestin-CreERT2 allele did not alter ABR threshold (Nptn+/+;Prestin-CreERT2+;tamoxifen, n = 3). Moreover, mice carrying both alleles, but dosed with vehicle (Nptnfl/fl;Prestin-CreERT2+;vehicle, n = 4) also had normal ABR thresholds. Data are mean ± S.D. (DOCX) [file pgen.1009937.s001.docx]

**Supporting Information**

***Neuroplastin* genetically interacts with *Cadherin 23* and the encoded isoform Np55 is sufficient for cochlear hair cell function and hearing**

Sherylanne Newton^1^, Fanbo Kong^2^, Adam J Carlton^2^, Carlos Aguilar^1^, Andrew Parker^1^, Gemma F Codner^3^, Lydia Teboul^3^, Sara Wells^3^, Steve DM Brown^1^, Walter Marcotti^2,4^ & Michael R Bowl^1,5*^

^1^Mammalian Genetics Unit, MRC Harwell Institute, Harwell Oxford, UK.

^2^School of Sciences, University of Sheffield, Sheffield, UK.

^3^Mary Lyon Centre, MRC Harwell Institute, Harwell Oxford, UK.

^4^Sheffield Neuroscience Institute, University of Sheffield, Sheffield, UK.

^5^UCL Ear Institute, University College London, London, UK


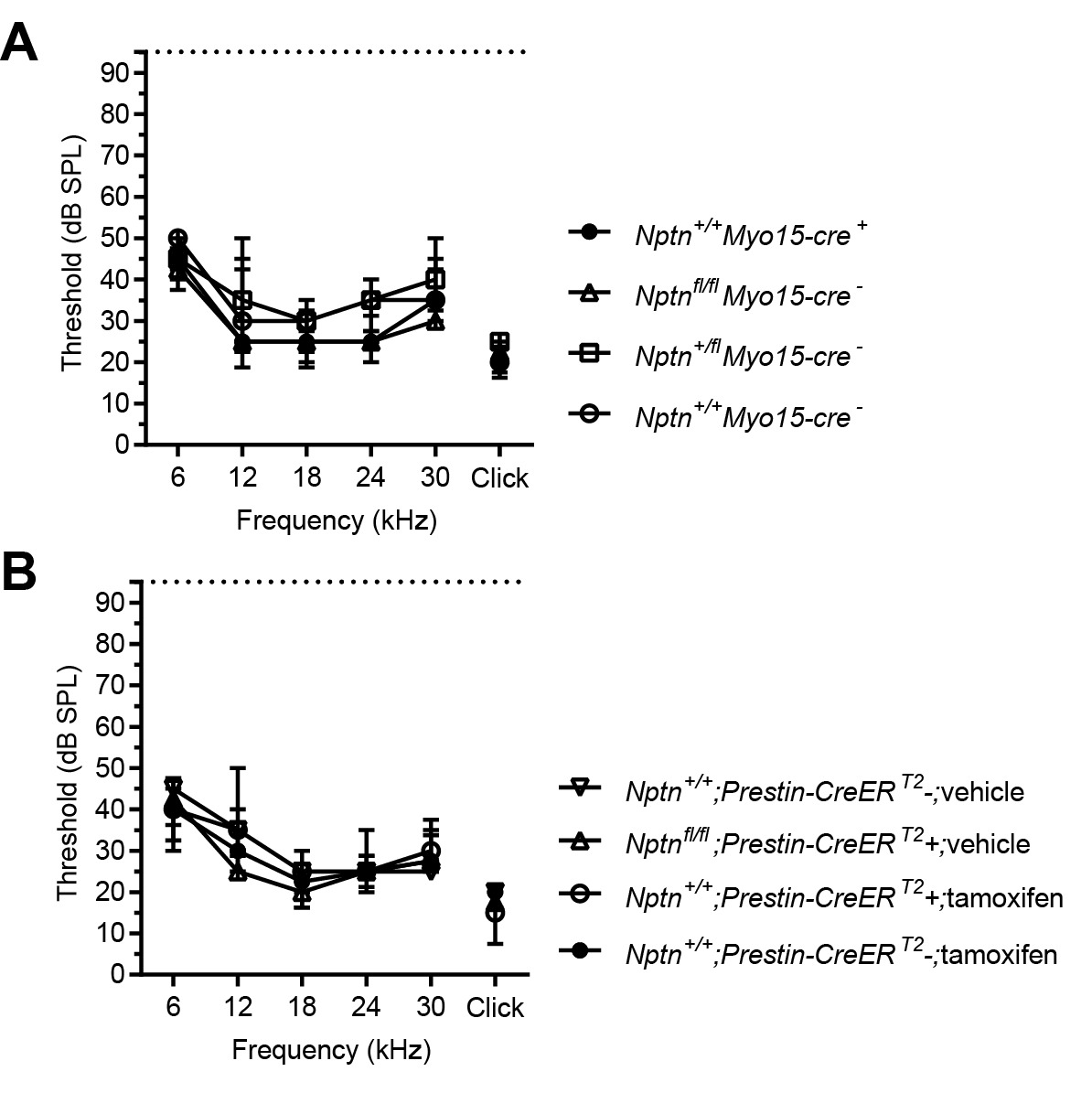


**S1 Fig. ABR data from the conditional knockout genotype control groups.  (A)** Control data from *Myo15-cre* conditional cross compared alongside wild type *Nptn^+/+^;Myo15-cre^-^* mice (n = 5). Presence of the either the *Myo15-cre* allele (*Nptn^fl/fl^;Myo15-cre^+^*, n = 5), or the floxed *Nptn* allele (*Nptn^+/fl^;Myo15-cre^-^*, n = 7 or *Nptn^fl/fl^;Myo15-cre^-^*, n = 6) did not affect ABR thresholds. **(B)** Control data from the Prestin-CreER^T2^ conditional cross compared alongside wild type *Nptn^+/+^;Prestin-CreER^T2^-* with (n = 4) or without tamoxifen (n = 3). Mice dosed with tamoxifen had comparable ABR thresholds to those dosed with vehicle only. Presence of the Prestin-CreER^T2^ allele did not alter ABR threshold (*Nptn^+/+^;Prestin-CreER^T2^+*;tamoxifen, n = 3). Moreover, mice carrying both alleles, but dosed with vehicle (*Nptn^fl/fl^;Prestin-CreER^T2^+*;vehicle, n = 4) also had normal ABR thresholds. Data are mean ± S.D.
